# Facile Synthesis of Wormhole-Like Mesoporous Tin Oxide via Evaporation-Induced Self-Assembly and the Enhanced Gas-Sensing Properties

**DOI:** 10.1186/s11671-018-2434-4

**Published:** 2018-01-11

**Authors:** Xiaoyu Li, Kang Peng, Yewei Dou, Jiasheng Chen, Yue Zhang, Gai An

**Affiliations:** 1 School of Materials Science and Engineering, Chang’an University, Xi’an, 710064 China; 2 State Key Laboratory for Mechanical Behavior of Materials, Xi’an Jiaotong University, Xi’an, 710049 China

**Keywords:** Evaporation-induced self-assembly (EISA) method, Mesoporous tin oxide, Semiconductor gas sensor, Gas-sensing properties

## Abstract

Wormhole-like mesoporous tin oxide was synthesized via a facile evaporation-induced self-assembly (EISA) method, and the gas-sensing properties were evaluated for different target gases. The effect of calcination temperature on gas-sensing properties of mesoporous tin oxide was investigated. The results demonstrate that the mesoporous tin oxide sensor calcined at 400 °C exhibits remarkable selectivity to ethanol vapors comparison with other target gases and has a good performance in the operating temperature and response/recovery time. This might be attributed to their high specific surface area and porous structure, which can provide more active sites and generate more chemisorbed oxygen spices to promote the diffusion and adsorption of gas molecules on the surface of the gas-sensing material. A possible formation mechanism of the mesoporous tin oxide and the enhanced gas-sensing mechanism are proposed. The mesoporous tin oxide shows prospective detecting application in the gas sensor fields.

## Background

Among semiconducting metal oxides, tin dioxide (SnO_2_), a wide band gap semiconductor (3.6 eV) with a rutile-type crystal structure, has been attracting much attention for various potential applications in the fields of anode materials of lithium-ion batteries [1], dye-sensitized solar cells [2], photocatalysis [3–5], conductive materials [6], and gas sensors [7] owing to its large band gap, nonstoichiometric nature, excellent electronic mobility, and stability. Nowadays, gas sensors are playing very important roles in the monitoring of environmental pollution [8], indoor air quality, public health, non-invasive disease diagnosis, and industrial applications. Many semiconducting metal oxides like ZnO [9], Co_3_O_4_ [10], WO_3_ [11–15], NiO [16, 17], and SnO_2_ [18–23] have been used for gas-sensing applications because of the excellent response, high sensitivity, good reliability, and low cost. Among them, SnO_2_ has been extensively investigated for gas sensors with a great sensitivity toward several gases, including acetone [24], nitrogen dioxide [25], toluene [26], ethanol [27], formaldehyde [28, 29], and methanol [30].

The properties of SnO_2_ directly depend on its structural and morphological state, such as the phase, particle size, and band gap. Therefore, many efforts were made to synthesize SnO_2_ into useful nanostructured morphologies to tailor its chemical and physical properties [17, 31, 32]. So, various SnO_2_ nanostructures with different morphologies have been obtained, which exhibited good sensing properties to many test gases. Meanwhile, SnO_2_ with mesoporous structure possesses high specific surface area and narrow pore size distribution, which can provide more in-situ active sites for superior interaction of SnO_2_ powders with analyte gas and easy gas diffusion into the porous sensing layers; it could further enhance the gas-sensing properties. Mesoporous SnO_2_ has been previously prepared through various methods including sol-gel and sonochemical methods utilizing supramolecular templates. However, the literatures relating to the preparation of SnO_2_ indicate that a simple and economic method to synthesize mesoporous SnO_2_ still poses a challenge and further improvement is necessary. Furthermore, evaporation-induced self-assembly is a pretty effective method for the synthesis of porous nanocrystals and has the advantages of homogeneous pore sizes, controllable morphologies, and mild reaction conditions [33, 34].

In this paper, a facile evaporation-induced self-assembly process was employed to synthesize SnO_2_ mesostructure under mild conditions for effective gas sensor application. The microstructure, morphology, and the sensing properties of the mesoporous SnO_2_ were systematically investigated. The test results about gas-sensing properties showed the as-prepared mesoporous SnO_2_ had a good sensitivity at an appropriate operating temperature, and the enhanced gas-sensing properties were closely related to their interconnected pores and exposed facets. Furthermore, the possible mechanism of enhanced gas-sensing properties was also discussed.

## Methods

All chemicals used in the experiments were analytical-grade reagents purchased from Sinopharm Chemical Reagent Co. Ltd. and used without further purification. In a typical procedure, 0.42 g SnCl_4_·5H_2_O and 0.336 g citric acid were first dissolved in 10 mL of deionized water. 0.144 g of structure-directing agent (template) (EO)_20_(PO)_70_(EO)_20_ (P123) was dissolved in 10 mL ethanol, and 1 mL of nitric acid was added as a condensation inhibitor. P123 solution was then added into the tin solution with vigorous stirring. The formed mixture was covered with PE film, stirred at 60 °C in water bath for 2 h, and then put into a drying oven at 60 °C to undergo solvent evaporation process. The as-formed solid was calcined in air for 3 h to remove the template and finally produce the mesoporous SnO_2_. The mesoporous SnO_2_ calcined at 350, 400, and 450 °C were named SnO_2_-350 °C, SnO_2_-400 °C, and SnO_2_-450 °C, respectively.

The phase analysis was performed at the D/MAX2550VB^+^ X-ray diffractometer with an acceleration voltage of 40 kV and an emission current of 300 mA, Cu Kα radiation (*λ* = 1.5405 Å) as radiation source, and graphite as monochromator; 2*θ* ranged from 0.5° to 80° was detected at a scanning rate of 0.02 °/s. Transmission electron spectroscopy (TEM) and high-resolution transmission electron microscopy (HRTEM) images of the products were taken by a Tecnai G^2^-20ST electron microscopy at 220 kV. The N_2_ adsorption-desorption isotherms were recorded at 77 K and analyzed using an ASAP 2020 Surface Area analyzer. The specific surface areas were calculated using the Brunnauer-Emmett-Teller (BET) equation, and estimates of the pore size distributions were deduced by means of Barrett-Joyner-Halenda (BJH) methods. Fourier-transform infrared (FTIR) spectra of the samples were recorded on a Nicolet Nexus 670 FTIR spectrophotometer using KBr pellets, and the mixture was pressed into a pellet for IR measurement. The photoluminescence (PL) spectrum was measured on a HITACHI FL-4500 at room temperature using a Xe lamp with a wavelength of 310 nm as the excitation source.

Firstly, the powders of mesoporous SnO_2_ were mixed with terpineol saturated with methylcellulose to form diluted slurry. Then, the slurry was coated onto an alumina ceramic tube which was printed with a pair of gold electrodes and four Pt wires. After being dried under ambient conditions, the ceramic tube was heated at 350 °C for 3 h. Finally, a small Ni-Cr alloy coil was inserted into the tube as a heater to provide the operating temperature.

The gas-sensing test was performed on a WS-30A system (Weisheng Electronics Co., Ltd., China). Before the measurements, the device was aged at 350 °C for 48 h in air to improve stability. The response was defined as Ra/Rg, where Ra and Rg were the resistances of the sensor exposed in air and in reducing atmosphere, respectively. The response and recovery times were defined as the time taken by the sensor to achieve 90% of the total resistance change in the case of adsorption and desorption, respectively. The humidity-sensing properties of mesoporous SnO_2_ sensors were studied at the optimum operating temperature under four different relative humidity (RH) (24, 43, 75, and 97%) using saturated solutions of CH_3_COOK, K_2_CO_3_, NaCl, and K_2_SO_4_, respectively. The testing principle of the gas sensors was similar to that described in the literature [21].

## Results and Discussion

**Fig. 1:**
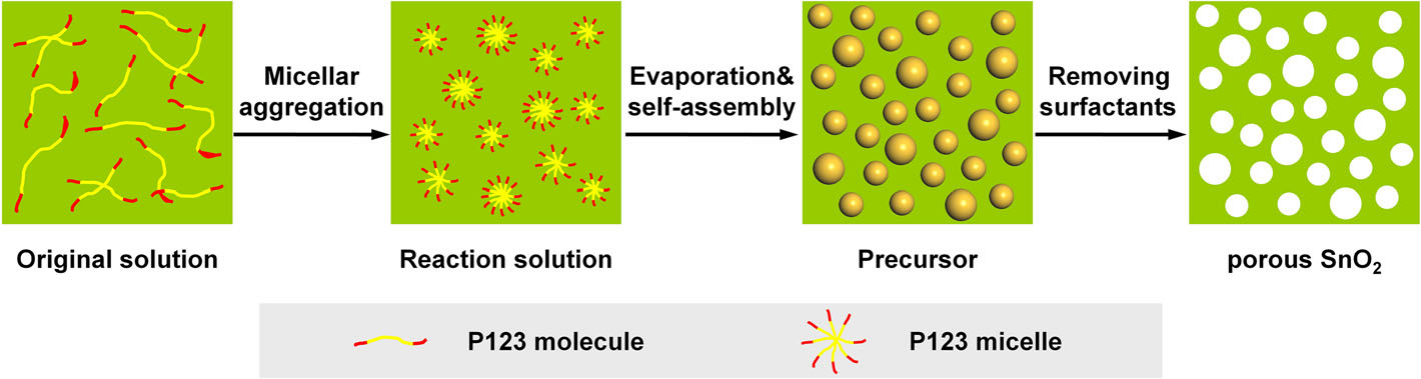
Schematic illustration of the synthetic procedure of mesoporous SnO_2_ powders

As illustrated in Fig. 1, mesoporous SnO_2_ powders were prepared through micellar aggregation, evaporation, self-assembly, and surfactants removal. Firstly, the tin species and P123 molecules were evenly mixed to form the original solution. The P123 served as a kind of structure-directing agent in the experiment, which subsequently assembled into micelles as liquid crystal mesophase. Under solvothermal conditions, P123 micelles could be adsorbed on the surfaces of Sn(OH)_4_ during slow evaporation progress in the solution or SnO_2_ by weak coordination bonds to form crown-ether-type complex intermediates that inhibit the growth of the SnO_2_ particles [35]. As a result, uniform SnO_2_ nanocrystals were obtained. Through the induced self-assembly of these particles and removal of the surfactant by a simple thermal treatment, the mesoporous-structured SnO_2_ was successfully obtained, which was responsible for high surface area and pore volume.

**Fig. 2:**
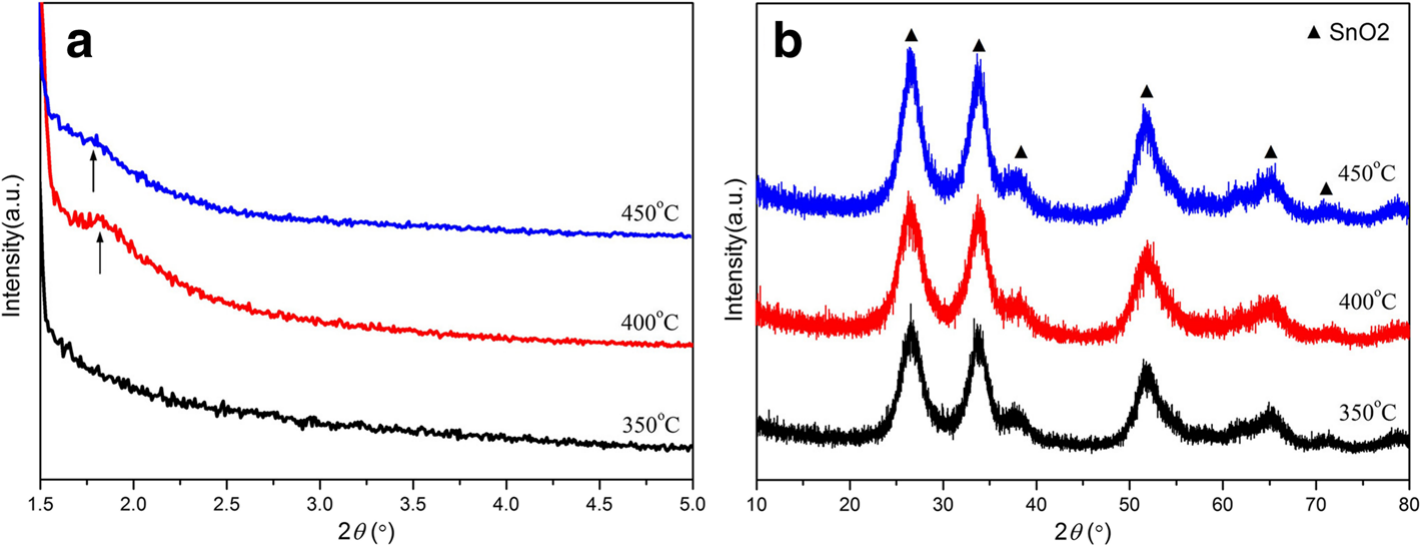
**a** Small-angle and **b** wide-angle XRD patterns of mesoporous SnO_2_ powders

The crystal structures of as-synthesized mesoporous SnO_2_ samples with different calcined temperatures were investigated by XRD measurements, and their patterns are shown in Fig. 2. The formation of the mesostructure was confirmed by small-angle XRD patterns (Fig. 2a). Sample SnO_2_-400 °C shows a stronger diffraction peak around 1.7°, characteristic of the mesoporous structure, while sample SnO_2_-350 °C shows no characteristic mesoporous peak. The sample SnO_2_-450 °C exhibits a relatively weaker and broader diffraction peak, which reveals that higher calcination temperature may result in the collapse of the mesostructure and the reduction of the corresponding diffraction peak. Figure 2b indicates the corresponding wide-angle XRD patterns of mesoporous SnO_2_ calcined at different temperatures. All the diffraction peaks are indexed to the tetragonal rutile structure of SnO_2_ (JCPDS card No. 41-1445) [36]. The diffraction peaks at 26.7°, 33.9°, and 52.0° can be indexed as the (110), (101), and (211) lattice planes, respectively. Furthermore, the increased intensity of SnO_2_ reflections for a higher calcined temperature indicates better crystallinity. The highly broadened peaks indicate that the SnO_2_ powders are composed of small-sized crystallites, which agrees well with the TEM results.

**Fig. 3:**
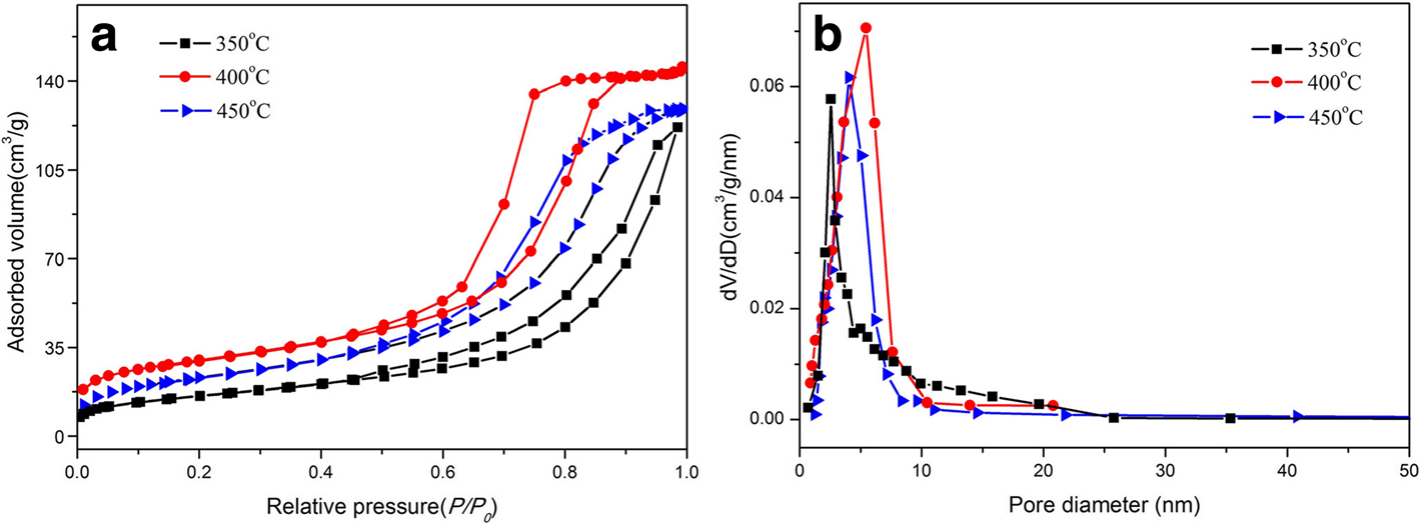
**a** Nitrogen adsorption-desorption isotherms. **b** Corresponding BJH pore size distribution curves of mesoporous SnO_2_ powders

The textural properties and pore structures of different samples were measured by the N_2_ adsorption/desorption isotherm. The N_2_ adsorption/desorption isotherm curves of mesoporous SnO_2_ calcined at different temperatures (Fig. 3a) exhibit a type IV adsorption branch with a distinct type *H3* hysteresis loop, and this type of isotherm is a typical characteristic of mesoporous structures [37–41]. The result is further confirmed by the corresponding BJH pore size distributions (Fig. 3b). The Brunauer-Emmett-Teller (BET)-specific surface areas (*S*_BET_) of SnO_2_-350 °C, SnO_2_-400 °C, and SnO_2_-450 °C were calculated to be 281, 356, and 307 m^2^/g, without an obvious decrease with the increase of calcination temperature, indicating the good thermal stability of mesoporous SnO_2_ prepared by one-step evaporation-induced self-assembly (EISA) method. Meanwhile, the total pore volume (*V*_pores_) and average pore diameter (*d*_pores_) were, respectively, calculated to be 0.14, 0.28, and 0.22 cm^3^/g and 2.9, 5.3, and 4.7 nm (Table 1). It shows a slight increase in textural properties from 350 to 400 °C, which is attributed to the complete removal of organic template and possible interconnection of the pore systems, while the tiny decrease from 450 to 400 °C is due to the slight collapse of the mesostructure.

**Table 1:** The textural characteristics of all samples

Material	*S*_BET_ (m^2^/g)	*V*_pores_ (cm^3^/g)	*d*_pores_ (nm)
SnO_2_-350 °C	281	0.14	2.9
SnO_2_-400 °C	356	0.28	5.3
SnO_2_-450 °C	307	0.22	4.7

Notes: *S*_*BET*_ BET specific surface area, *V*_*pores*_ total pore volume, *d*_*pores*_ average pore diameter

**Fig. 4:**
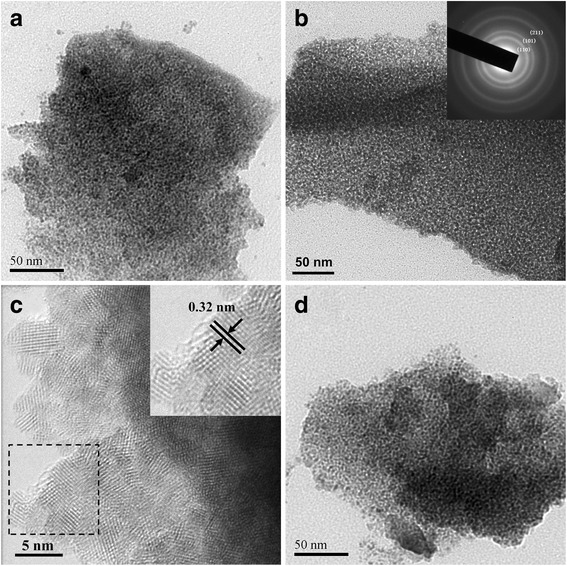
TEM images of **a** SnO_2_-350 °C, **b** SnO_2_-400 °C (inset is corresponding SAED pattern), and **d** SnO_2_-450 °C. **c** HRTEM image of SnO_2_-400 °C

The mesostructure of the samples could be confirmed by the TEM images. Typical TEM images of as-synthesized SnO_2_ samples calcined at different temperatures are shown in Fig. 4. It clearly displays the wormhole-like mesopores, which were formed by the aggregation of uniform nanoparticles. Such a pore structure is similar to that of the SnO_2_ samples fabricated by other researchers [42, 43]. The wormhole-like mesoporous structure can be enhanced by increasing the calcination temperature from 350 to 400 °C (Fig. 4a, b). The (selected area electron diffraction) SAED pattern of SnO_2_-400 °C (Fig. 4b) demonstrates the cassiterite polycrystalline structure, displaying three broad diffraction rings corresponding to (110), (101), and (221) reflections, respectively, which are well consistent with the XRD results. The HRTEM image of SnO_2_-400 °C (Fig. 4c) clearly showed its lattice fringe, and the lattice fringe spacing of SnO_2_-400 °C nanoparticles is 0.32 nm, which represented the (110) basal plane of SnO_2_ crystals. The mesoporous structure can retain well after calcination at 450 °C (Fig. 4d), indicating the excellent thermal stability of mesoporous SnO_2_.

**Fig. 5:**
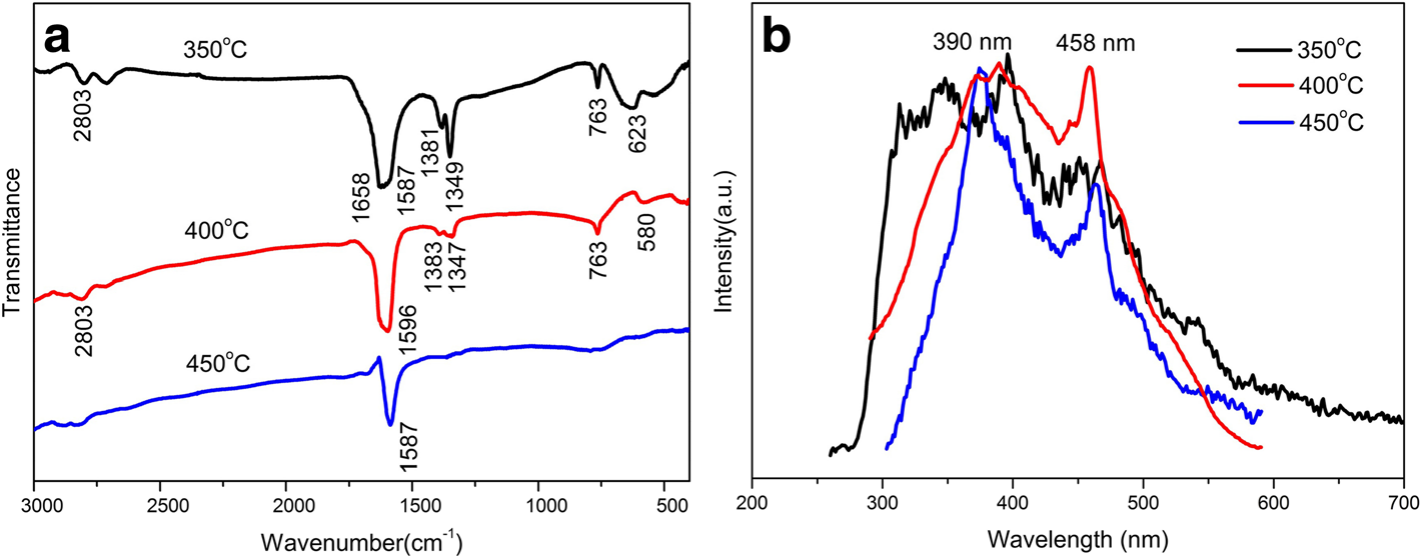
**a** FTIR spectra. **b** The photoluminescence spectra of mesoporous SnO_2_ samples (the excitation wavelength is 310 nm)

The FTIR spectra of different samples are shown in Fig. 5a. The characteristic stretching band at 1658 cm^−1^ is assigned to C=O group. The vibration bands at around 2803, 1381, and 1349 cm^−1^ are attributed to vibrations of CH_2_ species. The bands at around 763 and 623 cm^−1^ are assigned to different vibration modes of O–Sn–O and Sn–O–Sn groups. It indicated that there is a residual organic template in sample SnO_2_-350 °C. When the calcined temperature increases, the bands at 1658, 2803, 1381, and 1349 cm^−1^ are decreased because of the decomposition of the organic species. These bands disappeared for sample SnO_2_-450 °C, indicating that the surfactant template had been removed completely by calcination at 450 °C. Photoluminescence (PL) spectroscopy is a suitable technique to determine the crystalline quality and exciton fine structure [44]. Room temperature PL emission spectra were performed to investigate the optical properties of mesoporous SnO_2_. Figure 5b shows the PL emission spectra of the mesoporous SnO_2_ with different calcined temperatures, and the excitation wavelength was 310 nm. The samples calcined at 400 and 450 °C exhibit two main peaks in the emission spectra. One emission band is at about 390 nm, and the other one is at around 458 nm, indicating that increase temperature from 400 to 450 °C has little effect on optical properties of the samples, since the energy gap of bulk SnO_2_ was 3.62 eV. However, the peaks of SnO_2_-350 °C are noticeably more than those of the samples calcined at 400 and 450 °C, and this may be attributed to the residual organic template, which results in the surface of structural defects [45, 46]. The peak at 390 nm is independent of the concentration of oxygen vacancies, and it is from structural defects or luminescent centers, such as nanocrystals and defects of SnO_2_. The defects are mainly located on the surface of the nanostructures and could form a series of metastable energy levels within the band gap of mesoporous SnO_2_ by trapping electrons from the valence band. This makes a contribution to the luminescence or Sn interstitials formed during the evaporation-induced self-assembly process [47]. The peak at 458 nm is attributed to oxygen-related defects that have been introduced during the growth process [48]. The intensity of two emission bands increases with rising the calcined temperature, while the position of two emission bands has no obvious change.

**Fig. 6:**
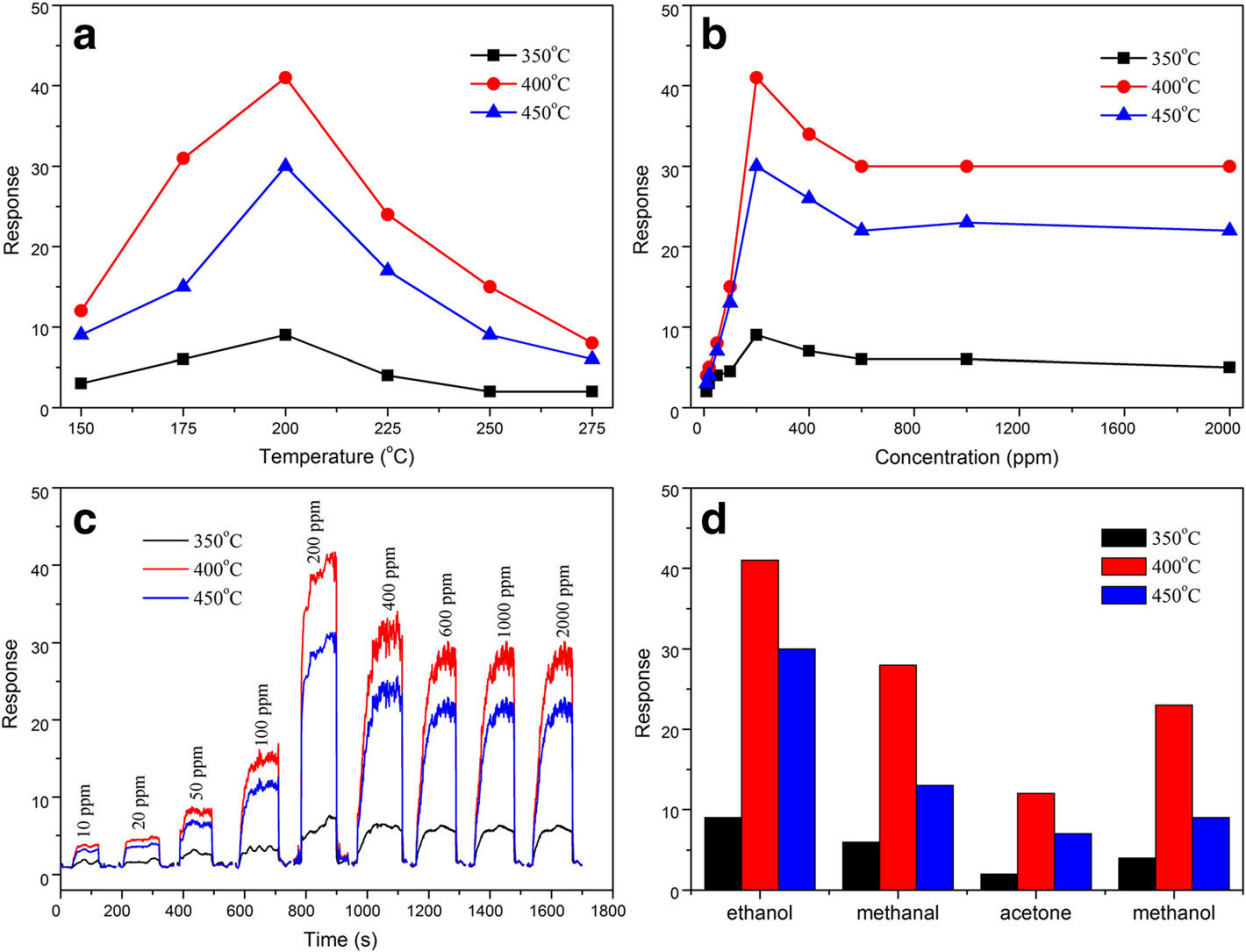
**a** Responses of the mesoporous SnO_2_ sensors to 200 ppm ethanol at different operating temperatures, **b** responses vs. ethanol concentration, and **c** response-recovery curve for the mesoporous SnO_2_ sensors (the operating temperature is 200 °C). **d** Gas responses of the mesoporous SnO_2_ sensors to 200 ppm of ethanol, methanal, methanol, and acetone operated at 200 °C

The gas-sensing properties of the mesoporous SnO_2_ sensors are shown in Fig. 6. Generally, the response of the gas sensors is influenced by its working temperature [49, 50]. Therefore, responses of the mesoporous SnO_2_ sensors with different calcined temperatures to 200 ppm ethanol at different operating temperatures (Fig. 6a) are investigated to determine the optimum working temperatures. It reveals that the responses of mesoporous SnO_2_ calcined at 400 °C remained highest at different operating temperatures, and yet, the responses are found to be reduced with an increase or decrease of the operating temperature. However, the responses of mesoporous SnO_2_ calcined at different temperatures have the similar trend, increasing firstly and decreasing later with rising operating temperature and the maximum occurs at 200 °C, indicating that the optimal working temperature of mesoporous SnO_2_ calcined at different temperatures to ethanol is 200 °C, and the following discussions are all based on the results measured at 200 °C.

Figure 6b shows the relationship curves tested at 200 °C between responses and ethanol concentration for the mesoporous SnO_2_ sensors calcined at different temperatures. It shows that the optimum ethanol concentration is 200 ppm for mesoporous SnO_2_ calcined at different temperatures. Mesoporous SnO_2_ calcined at 400 °C exhibits the highest response, and its response to 200 ppm ethanol reaches 41.6, which is much higher than that calcined at 350 and 450 °C. Figure 6c displays the response-recovery curves of the mesoporous SnO_2_ sensors for ethanol, which are tested under the same conditions (the operating temperature is 200 °C) in order to make a comparison. It revealed that the response speed of the SnO_2_-400 °C sensor is higher than SnO_2_-350 °C and SnO_2_-450 °C. The response and recovery time of the SnO_2_-400 °C sensor was 31 and 2 s, respectively. With the ethanol concentration increasing from 10 to 200 ppm, the gas-sensing properties curves show an increasing tendency, and the maximum response was 41.6 at 200 ppm. However, when the concentration of ethanol continuously increased to 400 ppm, their sensitivity are decreased and shows a leveling off from 400 to 2000 ppm, because the sensitivity of the sensors was saturated. Moreover, the responses of SnO_2_-350 °C and SnO_2_-450 °C show the similar varying tendency, but the responses are much lower than those of SnO_2_-400 °C. Selectivity is another important parameter to evaluate the sensing ability of a gas sensor [51, 52]. Figure 6d shows a bar graph of the mesoporous SnO_2_ sensors with different calcined temperatures to 200 ppm of ethanol, methanal, methanol, and acetone at the operating temperature of 200 °C. As shown in Fig. 6d, the sensors exhibit the highest response to ethanol against other target gases. In addition, the sensors are less sensitive to acetone. Meanwhile, the response of the mesoporous SnO_2_ calcined at 350, 400, and 450 °C to 200 ppm of ethanol is 9.3, 41.6, and 30.5, respectively. It can also be observed that the responses of the SnO_2_-350 °C sensor to 200 ppm of ethanol, methanal, acetone, and methanol are less than 10 at 200 °C. These results demonstrate that the as-prepared mesoporous SnO_2_ sensors can selectively detect ethanol vapors with the interference of other gases and have a good performance in the operating temperature and response/recovery time.

**Fig. 7:**
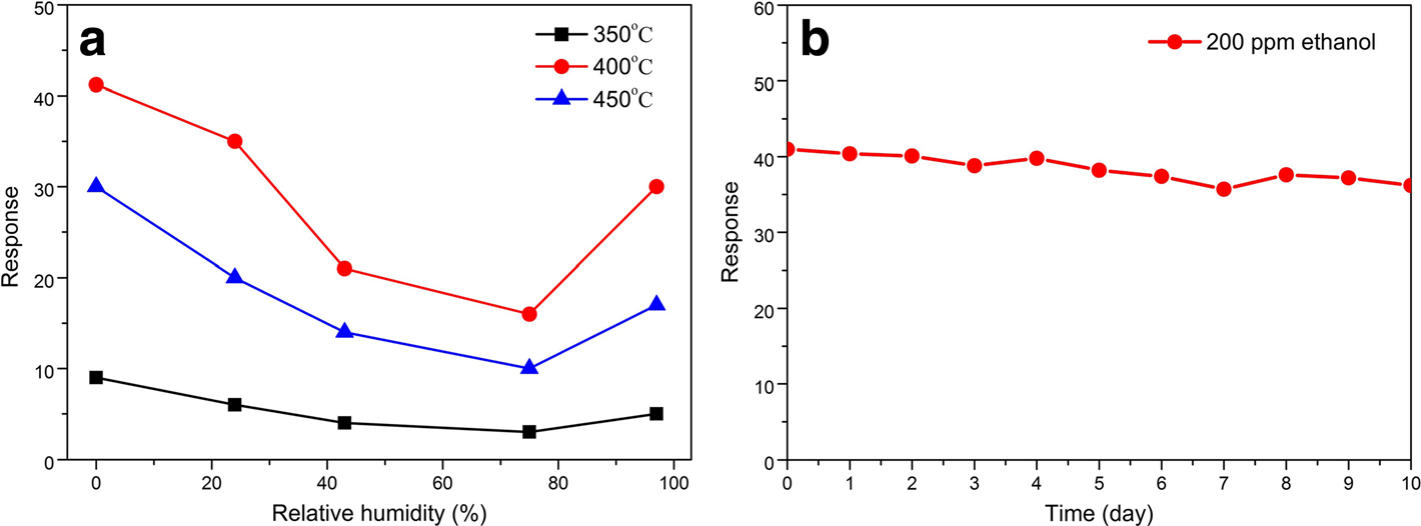
**a** Response of the mesoporous SnO_2_ sensors to 200 ppm of ethanol as a function of the relative humidity at 200 °C. **b** Long-term stability of the SnO_2_-400 °C sensor to 200 ppm ethanol at 200 °C

Relative humidity (RH) has an effect on the gas response of metal oxide-based gas sensors. Therefore, the influence of RH on this mesoporous SnO_2_ sensor was investigated, and the responses toward 200 ppm of ethanol under different RH are shown in Fig. 7a. It is clear that the responses decreased as the RH increased in comparison with dry conditions. Under 97% of RH, the response was about 17.2, 30.3, and 5.1 for the sensors SnO_2_-450 °C, SnO_2_-400 °C, and SnO_2_-350 °C, which were higher than the values found when RH was 43 and 75%. Moreover, the SnO_2_-400 °C demonstrated to be less affected by the presence of humidity, showing a lower decrease in ethanol response. The long-term stability of the SnO_2_-400 °C sensor was tested for 10 days under 200 ppm ethanol at the operating temperature of 200 °C, as shown in Fig. 7b. It is shown that the response changed every day, but the maximal deviations of the responses to ethanol are less than 10%. Clearly, the sensor based on the mesoporous SnO_2_-400 °C has an excellent long-term stability, which can be used as a promising candidate for practical gas-sensing applications.

Based on the results of gas-sensing properties for the mesoporous SnO_2_ sensors with different calcined temperatures, it was revealed that the mesoporous SnO_2_-400 °C sensor has the best comprehensive performance, which can be attributed to the high surface area and pore volume formed through the induction of self-assembly process. It shows a slight decrease in textural and gas-sensing properties when the calcined temperature rises from 400 to 450 °C, indicating that mesoporous SnO_2_ has good chemical stability and thermal stability. In addition, the decrease is due to the slight collapse of the mesostructure. The mesoporous SnO_2_-350 °C sensor has the worst overall performance, which is attributed to the channel plugging by the residual organic template. When the calcined temperature rose to 400 °C, the organic template was removed completely and may form the interconnected pore channels to enhance the gas-sensing performance further.

Some ethanol-sensing results of SnO_2_-based materials from the literature are summarized in Table 2. Our mesoporous SnO_2_ nanoparticles exhibited a better ethanol-sensing performance. The SnO_2_-400 °C shows the excellent response 41.6 at 200 °C for 200 ppm gaseous ethanol. The results indicate that the as-synthesized mesoporous SnO_2_ is a promising gas-sensing material for ethanol detection.

**Table 2:** Sensing performances of mesoporous SnO_2_ to ethanol in this work and previously reported sensing materials

Sensing material	Measuring range	Response	Reference
NiO/SnO_2_ thin film	100 ppm, 250 °C	7.9	[17]
SnO_2_ nanocrystals	100 ppm, 100 °C	11.2	[18]
SnO_2_–rGO	100 ppm, 25 °C	3.89	[22]
SnO_2_ bare	2 ppm, 150 °C	1.01	[25]
hollow SnO_2_ nanoparticles	100 ppm, 300 °C	63.4	[27]
SnO_2_ microtubes	50 ppm, 133 °C	3.4	[51]
mesoporous SnO_2_-350 °C	200 ppm, 200 °C	9.3	This work
mesoporous SnO_2_-400 °C	200 ppm, 200 °C	41.6	This work
mesoporous SnO_2_-450 °C	200 ppm, 200 °C	30.5	This work

**Fig. 8:**
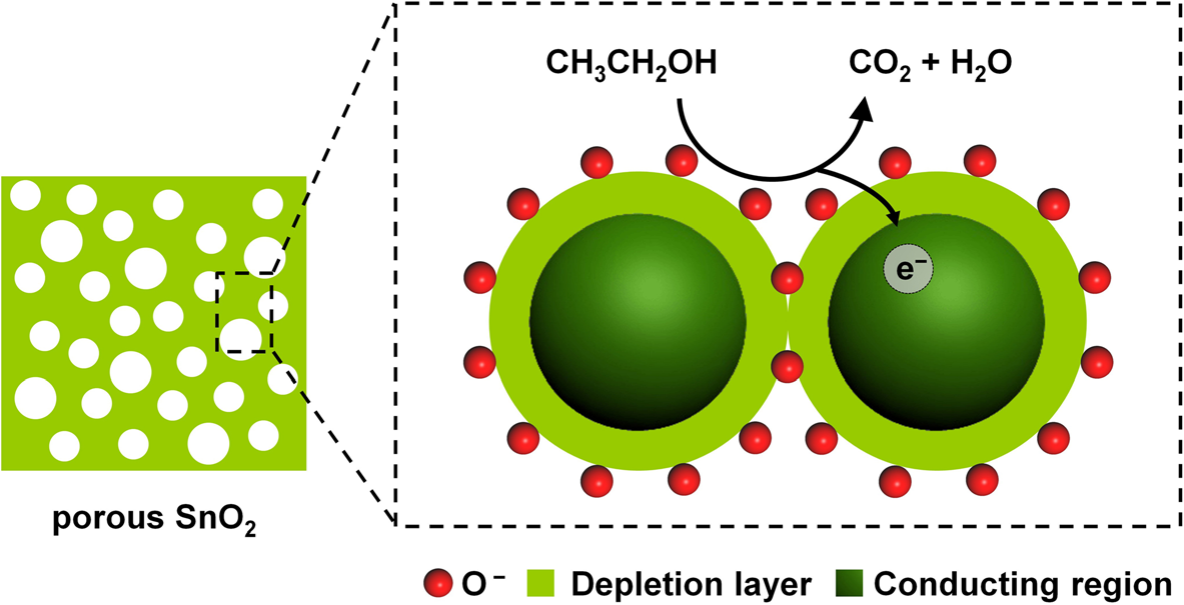
Schematic illustration of ethanol sensing mechanism of mesoporous SnO_2_

According to above results, we proposed the mechanism of the enhanced gas-sensing properties in Fig. 8. Generally, the narrow conducting channel in SnO_2_ nanocrystallines and high-potential barrier between SnO_2_ nanocrystallines makes the gas sensor show a high resistance value. Meanwhile, the accumulation of SnO_2_ nanoparticles hinders the effective diffusion of gases, which results in the degradation of gas-sensing properties. Therefore, improving the pore structure and increasing the specific surface area are efficient ways to improve the sensitivity of the sensor. At the micro scale, when the gas sensor was exposed to air, the oxygen species are ionosorbed on SnO_2_ surface (O_2_^−^, O^−^ or O^2−^) [18, 53] by trapping the electrons from the conduction band and created a depletion layer close to the particle surface. In dry air, O^−^ is the predominant ionosorbed oxygen species [27, 54]; therefore, a reaction between O^−^ species ionosorbed on mesoporous SnO_2_ nanoparticles and ethanol occurs. As a result, the electrons are released back to the conduction band of SnO_2_ and the O^−^ species are transformed into water and carbon dioxide. This results in a decrease in the depletion layer along with a resistance decrease. Therefore, in the experiments, mesoporous SnO_2_ with high specific surface area can provide more active sites and generate more chemisorbed oxygen spices on the surface, which increases the depletion layer of SnO_2_. Moreover, the porous structure and nano size of SnO_2_ particles allow an efficient diffusion of oxygen and test gas (ethanol) to active sites, which makes the sensor show a higher response to target gas (ethanol).

## Conclusions

In summary, the SnO_2_ with mesoporous nanostructures were successfully fabricated by means of evaporation-induced self-assembly technique, using triblock copolymer P123 as the template and tin (IV) chloride pentahydrate as the metal precursor, and calcined at different temperatures. The results revealed that the mesoporous SnO_2_ have good chemical and thermal stability. In the gas-sensing studies, the mesoporous SnO_2_ exhibited enhanced gas-sensing properties, such as fast response/recovery time, high sensitivity, and good sensing selectivity to ethanol. Mesoporous SnO_2_ calcined at 400 °C exhibits the highest response, and its response to 200 ppm ethanol reaches 41.6. This might be attributed to their high specific surface area and interconnected pores structure, which can provide more active sites and generate more chemisorbed oxygen spices to promote the diffusion of ethanol molecules and their adsorption on the surface of the SnO_2_. We believe that the mesoporous SnO_2_ could have excellent detecting application in the field of pollution detecting, medical diagnosis, and industrial security.

## Abbreviations

BET: Brunauer-Emmet-Teller
BJH: Barrett-Joyner-Halenda
*d*_pores_: Average pore diameter
EISA: Evaporation-induced self-assembly
JCPDS: Joint Committee Powder Diffraction Standards
P123: (EO)_20_(PO)_70_(EO)_20_
PL: Photoluminescence
*S*_BET_: Specific surface areas
SnO_2_: Tin oxide
TEM: Tansmission electron microscopy
*V*_pores_: Total pore volume
XRD: X-ray diffraction
